# Paclitaxel, Imatinib and 5-Fluorouracil Increase the Unbound Fraction of Flucloxacillin In Vitro

**DOI:** 10.3390/antibiotics9060309

**Published:** 2020-06-08

**Authors:** Maximilian Stolte, Weaam Ali, Janne Jänis, Andre’ Gessner, Nahed El-Najjar

**Affiliations:** 1 Institute of Clinical Microbiology and Hygiene, University Hospital Regensburg, 93053 Regensburg, Germany; maximilianstolte@gmx.de (M.S.); weaam.abdulrahman@azhar.edu.eg (W.A.); Andre.Gessner@klinik.uni-regensburg.de (A.G.); 2 Department of Chemistry, University of Eastern Finland, FI-80100 Joensuu, Finland; janne.janis@uef.fi

**Keywords:** albumin, α-1-acid glycoprotein, drug-drug interactions, anti-infective agents, ultrafiltration, cancer

## Abstract

Flucloxacillin (FLU), an isoxazolyl penicillin, is widely used for the treatment of different bacterial infections in intensive care units (ICU). Being highly bound to plasma proteins, FLU is prone to drug-drug interactions (DDI) when administered concurrently with other drugs. As FLU is binding to both Sudlow’s site I and site II of human serum albumin (HSA), competitive and allosteric interactions with other drugs, highly bound to the same sites, seem conceivable. Knowledge about interaction(s) of FLU with the widely used anticancer agents paclitaxel (PAC), imatinib (IMA), and 5-fluorouracil (5-FU is scarce. The effects of the selected anticancer agents on the unbound fraction of FLU were evaluated in pooled plasma as well as in HSA and α-1-acid glycoprotein (AGP) samples, the second major drug carrier in plasma. FLU levels in spiked samples were analyzed by LC-MS/MS after ultrafiltration. Significant increase in FLU unbound fraction was observed when in combination with PAC and IMA and to a lesser extent with 5-FU. Furthermore, significant binding of FLU to AGP was observed. Collectively, this is the first study showing the binding of FLU to AGP as well as demonstrating a significant DDI between PAC/IMA/5-FU and FLU.

## 1. Introduction

Most drugs travel the circulation system bound to plasma proteins and only free drugs are able to reach the sites of action to induce the desired pharmacological effects [1]. The drugs are, therefore, in an equilibrium state between bound and unbound fractions and the carrier proteins act as a reservoir slowly releasing the drugs to maintain the established equilibrium. Binding of drugs to plasma proteins prevents their fast metabolism as well as the attainment of toxic levels in the body [1]. Cancer patients in ICU often suffer from kidney and/or liver impairments as well as from hypoalbuminemia, all of which affect the pharmacokinetic/pharmacodynamics (PK/PD) target attainment of the antimicrobial therapy. DDI can affect the unbound fraction of the antimicrobial therapy by affecting the metabolism and excretion of the antimicrobial agent used. Consequently, DDI caused by altered protein binding that affect the unbound fraction of a drug can have a significant impact on the drug’s effect as even small changes in the percentage of binding can already increase the unbound fraction by several fold [1]. This is critical in the case of antimicrobial agents as any change affecting their PK/PD target attainment [2] can alter the clinical outcome of the prescribed therapy. For instance, an increase in the concentration of the antimicrobial agents can be associated with toxicity while a decrease in the concentration can result in resistance [3]. Unfortunately, DDI can play a significant role in the increase in mortality and morbidity of cancer patients [4]. This is not surprising as the complicated health situation of cancer patients necessitates in many instances the concomitant administration of a wide range of medications such as immunosuppressant and anti-infective agents along with antineoplastic agents [5]. A study by Poulikakos et al., showed a mortality rate of 100% of cancer patients after an influenza infection even though a proper treatment was received [6]. The reasons for this outcome are not known, but may have been caused by toxicity or under-dosing of the anticancer/anti-infective agents. The likelihood of DDI between antibiotics and antineoplastic agents is not unexpected as both agents are characterized by their narrow therapeutic index and high level of inter- and intra-individual variability [7,8]. Possible DDI between antibiotics and anticancer agents are already known, and can be manifested at the PK level where the absorption, distribution, metabolism, elimination of the drug itself or a combination of drugs is affected, or at the PD level resulting in synergistic, additive, or antagonistic effects [7]. While Methotrexate, an anti-folate agent effective against different types of tumors, is extensively studied when it comes to antibiotic-antineoplastic interaction [9,10,11], data on drug displacement of broad-spectrum antibiotics by anticancer agents are scarce. Direct changes in protein binding caused by competition at the binding site can easily alter the PK/PD profile of the administered drugs, especially the plasma clearance. Although DDI caused by changes in protein binding is not clinically important for most drugs, it might have severe clinical consequences for highly bound ones (≥90%) [12]. FLU, used for the treatment of many infections that are caused by gram-positive species i.e., *Streptococci* and penicillinase-producing *Staphylococci* i.e., methicillin-susceptible *Staphylococcus aureus*, is known for its high binding to plasma proteins (95–97%) in healthy patients [13]. HSA, known as a high capacity low affinity carrier and which represents nearly 52–60% of the proteins in human blood serum, is the most important protein for the transport of drugs in the body [14]. It is synthesized in the liver, has a serum concentration that ranges from 35 to 50 g/L, a molecular mass of 66 kDa, and a serum half-life time of roughly 19–20 days [14]. AGP, one of the most important glycoproteins in human plasma, is known as a high affinity low capacity protein that binds both endogenous and exogenous ligands and transport them to their target sites [15]. It is mainly produced in the liver and has an average plasma level of 0.5–1 g/L in healthy adults [16]. As it has only one drug-binding site, drugs binding primarily to AGP are susceptible to displacement by other AGP ligands [16]. Hypoalbuminemia encountered in 40–50% of ICU patients, the systemic inflammatory response syndrome, the use of inotropes and fluid resuscitation can be the cause for variations in the PK/PD [17] of FLU [13]. Nevertheless, further alterations in the protein binding, due to DDI, could also have serious implications for the clinical outcome. The fact that renal clearance and multiple half-lives within a dosing interval of highly bound drugs are most likely affected by changes in the protein binding [18], makes FLU highly prone to DDI. Consequently, this study aimed to evaluate in vitro DDI between FLU and three anticancer agents (PAC, IMA, and 5-FU) widely used against different types of cancer and which bind to the same binding sites as FLU. The unbound fraction of FLU was determined, in the presence and absence of the anticancer agents, by ultrafiltration followed by LC-MS/MS analysis. This study shows for the first time the significant binding of FLU to AGP as well as a significant increase in FLU’s free levels in the presence of IMA, PAC, or 5-FU, which can be clinically relevant following their co-administration.

## 2. Results

### 2.1. Determination of Protein Binding of FLU

Prior to the evaluation of interaction between FLU and the selected anticancer drugs (5-FU, PAC and IMA), we aimed to simulate using our experimental procedure the physiological conditions and determine the protein binding of FLU alone when tested at concentrations expected in patients [19]. To achieve this aim, spiked plasma samples were incubated at 37 °C for 30 min, processed, and analyzed as previously described. The obtained data shows that FLU has a protein binding of 99.02% to 99.41%.

### 2.2. Interaction between 5-FU, PAC, IMA and FLU in Pooled Plasma

The interactions between FLU and the anticancer drugs: IMA, PAC, and 5-FU were tested at two different levels of FLU [low (0.2 µg/mL), which fits within its range of minimum inhibitory concentration (MIC) against different bacterial strains [20] and high (20 µg/mL), the 100-fold MIC still expected within the dosing interval reported in patients]. The anticancer agents, which were supplemented to pooled plasma spiked with FLU, were tested at concentrations that fit with their therapeutic concentrations in patients [21,22,23,24,25,26,27]. In comparison to plasma samples spiked with FLU alone, all samples containing either of the anticancer agents and FLU showed increased free levels of FLU (Table 1, Table 2 and Table 3).

#### 2.2.1. Interactions between FLU and PAC

**Figure 1:**
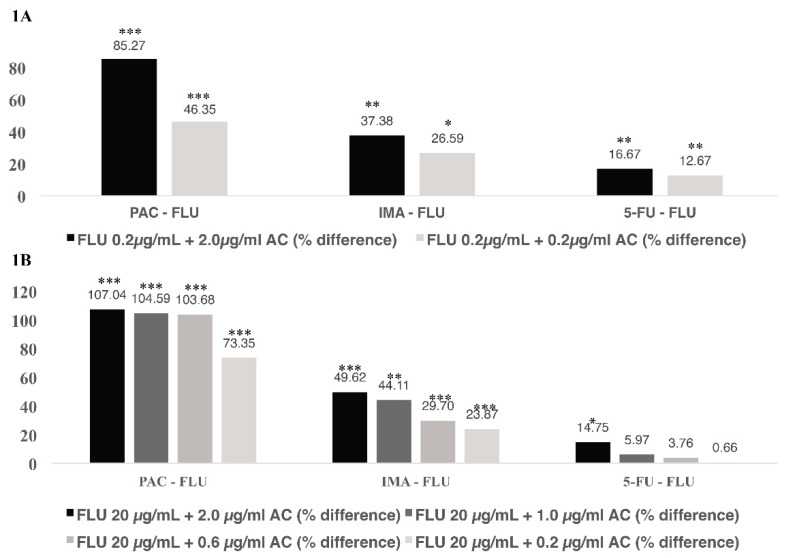
Comparison of the percent increase of FLU unbound fraction in mixture with IMA, PAC and 5-FU at 0.2 µg/mL (**A**) and 20 µg/mL (**B**). Percent difference calculated with respective to the percent-unbound values of FLU alone at 20 µg/mL and 0.2 µg/mL. AC = anticancer agent. * *p* < 0.05, ** *p* < 0.01, *** *p* < 0.001 with respect to FLU alone.

PAC increases the unbound fraction of FLU (Table 1). Samples containing FLU and PAC at 1:1 ratio already showed increased levels of free FLU while a higher increase was seen in samples containing FLU (0.2 µg/mL) and PAC (2.0 µg/mL) (1:10 ratio of FLU:PAC, Figure 1A), probably due to higher levels of the anticancer agent.

PAC (0.2–2 µg/mL) also resulted in a significant increase in the free fraction of FLU (20 µg/mL). While a PAC concentration of 0.2 µg/mL already increased the unbound concentration of FLU significantly, the increase was around 2-fold at concentrations of 0.6 µg/mL and higher (Figure 1B). Increasing the concentration of PAC (up to 2.0 µg/mL) did not result in a significantly higher increase in unbound FLU (Figure 1B).

#### 2.2.2. Interaction between FLU and IMA

IMA increases the unbound fraction of FLU (Table 2). When tested at 1:1 and 1:10 ratio (FLU:IMA), FLU unbound fraction were increased, respectively, by around 1.3-fold (Figure 1A). At higher levels of FLU (20 µg/mL), a significant increase of FLU unbound fraction was seen at all IMA concentrations. The highest increase was seen at 2.0 µg/mL, where free levels of FLU were increased by around 1.5-fold (Figure 1B).

#### 2.2.3. Interaction between FLU and 5-FU

Only minor increase in FLU was seen in presence of 5-FU. At low FLU concentrations (0.2 µg/mL), the unbound concentration was only increased by 1.15-fold (Figure 1A). As expected the effect of 5-FU was even less at higher levels of FLU (20 µg/mL) (Figure 1B).

#### 2.2.4. Interactions of FLU with Warfarin and Diazepam in Pooled Plasma

Warfarin, a vitamin k antagonist and widely used anticoagulant, is a commonly used displacement marker for Sudlow’s site I on HSA [28], while diazepam, a benzodiazepine, is commonly used as a Sudlow’s site II displacement marker [29]. Therefore, the displacement of FLU was also tested in the presence of warfarin and diazepam at concentrations encountered in patients [30,31,32]. Pooled plasma spiked with FLU/warfarin or FLU/diazepam were analyzed as previously prescribed. Warfarin and diazepam tested at a concentration of 1.0 µg/mL significantly increased the unbound FLU (20 µg/mL) (Table 4).

### 2.3. Binding of FLU to HSA and AGP

#### 2.3.1. Binding of FLU to HSA at Physiological Levels

To evaluate the percentage of binding of FLU to HSA as well as to further confirm the interactions observed between FLU and the tested anticancer agents, FLU was spiked into samples containing physiological concentrations of HSA, which were set to 40 g/L. The percentage of binding of FLU to HSA (40 g/L) [33] ranged from 93.98% to 94.39%, indicating an unbound fraction of 5.61% to 6.02%, respectively (Table 5).

#### 2.3.2. Interactions between FLU and PAC, IMA and 5-FU at Physiological Levels of HSA

The displacement of FLU by the anticancer drugs, tested at 1.0 µg/mL, was evaluated in the presence of HSA (40 g/L). PAC increased by about 1.15-fold the unbound fraction of FLU tested at 0.2 µg/mL and 20 µg/mL (Table 5). Likewise, at physiological concentration of HSA, IMA also increased the unbound fraction of FLU (0.2 and 20 µg/mL) by around 1.2-fold (Table 5). The minimally higher effect observed with FLU (0.2 µg/mL) is probably due to the higher ratio of PAC and IMA to FLU compared to the samples containing FLU (20 µg/mL). Lower effect was seen when 5-FU is present. At 5-FU (1 µg/mL) and FLU (0.2 µg/mL) there was no significant difference in the unbound fraction of FLU in HSA solution and only a minimal increase was seen at 20 µg/mL of FLU (Table 5).

#### 2.3.3. Binding of FLU to AGP at Physiological Levels

**Figure 2:**
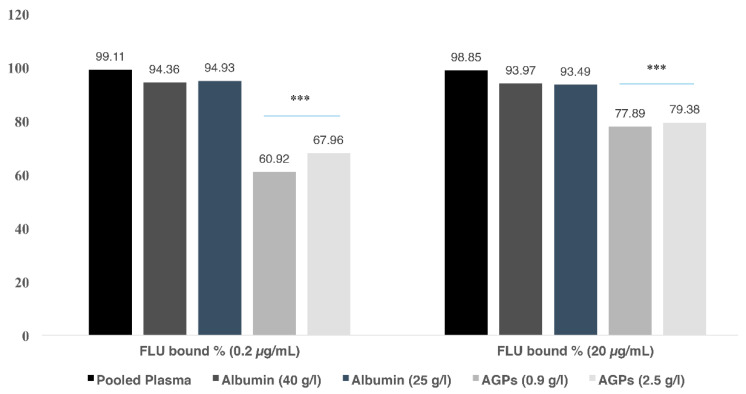
Comparison of the % binding of FLU to pooled plasma, albumin, and α-1-acid glycoprotein at physiological and pathophysiological concentrations. *** *p* < 0.001.

While FLU is known to bind to HSA, nothing is reported about its binding to AGP. In this part the binding of FLU to AGP was evaluated at physiological concentration (0.9 g/L). Interestingly, FLU alone showed a significant percentage of binding to AGP (Figure 2).

### 2.4. Interactions between FLU and IMA

As IMA is mainly binding to AGP, we also tested its interactions with FLU at physiological AGP concentrations (0.9 g/L). Interestingly, the increase in the unbound fraction of FLU at 20 µg/mL was not significant (Table 6), while it increased by 1.2-fold at a concentration of 0.2 µg/mL (Table 6). The increased effect is probably due to the much higher ratio of IMA (1 µg/mL) to FLU (0.2 µg/mL) (5:1) compared to FLU (20 µg/mL).

### 2.5. Binding of FLU to HSA at Pathophysiological Levels

Hypoalbuminemia, caused by several factors like renal and liver diseases, inflammatory diseases, surgery, burn and malnutrition [1], is common in 40–50% of ICU patients [13]. The effects of hypoalbuminemia on the PK of highly bound drugs have already been reported [13]. Therefore, to examine the differences in the protein binding of FLU and the DDI with IMA, PAC and 5-FU, the same displacement experiments were repeated at pathophysiological solution of HSA (25 g/L) [13]. The percent of binding of FLU in the presence of 25 g/L HSA ranged from 94.91% bound (0.2 µg/mL) to 93.49% (20 µg/mL) (Figure 2).

#### 2.5.1. Interactions between FLU and PAC, IMA, and 5-FU at Pathophysiological Levels of HSA

The displacement of FLU by the anticancer drugs at 1.0 µg/mL was tested in the presence of pathophysiological concentrations of HSA. An increase in FLU (20 µg/mL) unbound fraction was seen in samples containing pathophysiological HSA levels after addition of PAC (Table 5). The more pronounced effect in PAC-FLU (0.2 µg/mL) samples whereas the unbound fraction increased by 1.4-fold (Table 5), is probably caused by the higher ratio of PAC compared to FLU. IMA showed similar effect as with the physiological conditions as the increase of free FLU at 0.2 µg/mL and 20 µg/mL accounted for 1.2-fold (Table 5). 5-FU also only showed minor effect to the free fraction of FLU at HSA (25 g/L) at both concentrations (0.2 and 20 µg/mL) (Table 5).

#### 2.5.2. Binding of FLU to Pathophysiological Levels of AGP

The plasma levels of AGP, a positive acute phase protein, can increase 3- to 5-fold during different conditions such as in cancer, infections, trauma, and inflammation [16]. AGP was prepared at a concentration of 2.5 g/L to simulate the pathophysiological state of increased AGP levels in human plasma. AGP solution was spiked with FLU alone and the unbound FLU fraction was determined. The binding of FLU to AGP samples was 67.96% and 79.38% when FLU’ concentrations were 0.2 µg/mL and 20 µg/mL, respectively (Figure 2). The unbound fraction of FLU (20 µg/mL) slightly increased from 77.89% bound at physiological levels AGP (0.9 g/L) to 79.38% bound at pathophysiological AGP levels (2.5 g/L). Likewise, but a more distinct effect was seen with FLU (0.2 µg/mL) whereas the bound fraction increased from 60.92% at AGP (0.9 g/L) to 67.96% AGP (2.5 g/L) (Figure 2).

#### 2.5.3. Interactions between FLU and IMA at Pathophysiological Levels of AGP

The displacement of FLU by IMA (1.0 µg/mL) was further tested in the presence of AGP (2.5 g/L). In the presence of FLU (20 µg/mL), the increase in the unbound fraction of FLU seen after addition of IMA was minor (Table 6). Similar to the physiological conditions of AGP, the percent increase in unbound fraction of FLU (0.2 µg/mL) was much higher as it accounted for more than 1.2-fold (Table 6).

## 3. Discussion

Drugs bind usually, with different affinity and selectivity, to two major plasma proteins, namely HSA and AGP [27,33,34,35]. While the bound fraction serves as a reservoir, the unbound fraction distributes into the human body tissues and the extravascular space to induce the pharmacological effect [17,36]. Changes in the free fraction of highly bound drugs, due to DDI have significant impact on the drug’s effect and toxicity, as even small changes in the percentage of binding can already increase the unbound fraction by several folds. This in turn influences the drug’s renal clearance and the multiple half-lives within a dosing interval [18]. FLU, a broad-spectrum time dependent β-lactam antibiotic, is one of the most widely used antibiotics for the treatment of *Staphylococcus aureus* acquired in the ICU [37]. FLU is more than 95% bound to plasma proteins [13,17,38], with typical unbound fraction of 3–5% in patients with normal HSA (>42 g/L). Therefore, FLU is highly prone to DDI. As FLU is associated with liver damage and the development of jaundice [39], altered PK/PD could be dangerous for patients treated with FLU. An altered drug distribution, caused by the competition for plasma protein binding is one of the main reasons for DDI [12]. Only drugs with similar physicochemical properties and which are concurrently administered can compete with each other’s and with other endogenous substances for common or functionally linked binding sites [34]. Displacement interactions through direct competition or allosteric modification can lead to faster elimination or an increase in the pharmacological activity/toxicity of the displaced drug [1], which could be problematic for time-dependent antibiotics per se. For instance, it has been reported that the binding of a drug can induce three-dimensional structural changes in HSA, which have been shown to have unpredictable effects on the drug binding even if the changes are minor [1]. Allosteric activation, caused by the binding of molecules, serving as allosteric effectors, has already been described for HSA [40]. Particularly, interactions of FLU with HSA [41] and other drugs are previously reported. For instance, a decrease in the international normalized ratio was shown in several reports after FLU was added to warfarin therapy, probably caused by an induction of the hepatic CYP3A4 isoenzyme [42]. Further interactions with piperacillin [42,43], voriconazole [44,45], rifampicin [46], quinolones [47] and paracetamol [48] have been shown in different studies.

Although displacement interactions have been shown to be of little clinical value for healthy individuals, the situation is different for critically ill patients, especially those with liver or kidney impairment commonly encountered in the ICU [1]. The simultaneous administration, to ICU cancer patients, of antitumor agents and antibiotics [49], characterized by their narrow therapeutic index and inter- and intra- individual variability, makes them prone to DDI [50,51]. Data on drug displacement effects of anticancer agents on FLU is lacking. This study evaluated the displacement interaction of three anticancer agents (PAC, IMA, and 5-FU) on the unbound fraction of FLU in pooled plasma, as well as in HSA and AGP solutions. FLU binds to Sudlow’s site I and site II of HSA [16] and modifies residues in both hydrophobic pockets. PAC, a taxane anticancer agent, has a wide range of antitumor activities against ovarian, breast, gastric, prostrate, head and neck, and non-small-cell lung cancers [21]. PAC, a highly bound drug (90% bound [52]), binds primarily to the subdomains IIA and IIIA of Sudlow’s site I and site II of HSA [53]. When bound to Trp214 in subdomain IIA PAC causes changes in the protein conformation of the secondary structure and the local conformation of HSA [53]. IMA, a protein tyrosine kinase Bcr-Abl and c-KIT selective inhibitor, is used for the treatment of gastrointestinal stromal tumors and chronic myeloid leukemia [54,55]. IMA, which is highly bound to human plasma proteins (95%) [54], primarily to AGP, also shows binding affinity to subdomain IIA of Sudlow’s site I (FA1 and FA7) and subdomain IB of HSA [56,57]. 5-FU, uracil analogue, is widely used for the treatment of cancers such as colon, breast, and skin among others [58]. 5-FU binds to the subdomain IIA on Sudlow’s site I on HSA [59].

In this in vitro study, we evaluated first the binding of FLU to plasma proteins in pooled plasma and in solutions containing physiological levels of the two major drugs’ carriers in plasma (HSA and AGP) prior the evaluation of the displacing effect of PAC, IMA and 5-FU. The data shows that FLU has a protein binding ranging from 99.02 to 99.41%. The higher binding of FLU observed in this study, compared to other studies (93% [38], 95% [36], 95–97% [13]), can be attributed to the fact that most studies investigating the protein binding of FLU were in blood samples directly taken from patients after oral administration of FLU [13,38], while in this study FLU is spiked into pooled plasma samples obtained from healthy volunteers. In patients treated with FLU, other drugs can be concomitantly administered and can still be bound to the proteins affecting thus the percentage of binding of FLU. In addition, different information can be obtained if different settings for samples’ processing and analysis are used. For instance, the unbound concentration is often estimated rather than directly measured [60,61,62]. The percentage of binding of FLU to physiological levels of HSA (40 g/L) ranged from 93.98% to 94.39%. Considering that pooled plasma and HSA solution contains protein levels that exceeds those of FLU, the reason for the five times higher unbound fraction of FLU observed when in the presence of physiological HSA compared to the one in pooled plasma can be attributed to a potential increase in the affinity of the proteins in plasma to FLU. It has been shown that unbound fraction of a drug can be significantly altered in the absence of endogenous fatty acids per se. Furthermore, it is possible that plasma contains other carriers/binding partners that also binds FLU. It is known that FLU binds to HSA while nothing is known about its binding to AGP. Interestingly, this study shows for the first time that FLU binds significantly to physiological concentrations of AGP (0.9 g/L) [60.92% (0.2 µg/mL) and 77.89% (20 µg/mL) of FLU] (Figure 2). In opposition to the stable binding of FLU to HSA 94.39% (0.2 µg/mL FLU) and 93.98% (20 µg/mL FLU) the large variation observed in the binding of different concentration of FLU to AGP solutions merits further investigation. Nevertheless, the binding of FLU to AGP has clinical implications as suggests that HSA and AGP can compensate for each other for the binding of FLU in cases where significant changes affect their levels.

The displacement of FLU was assessed and significant increase in the unbound fraction of FLU has been shown in the presence of PAC, IMA, and to a lesser extent with 5-FU in pooled plasma and HSA samples (40 g/L), as well as in the presence of IMA in AGP samples (0.9 g/L).

The effect of PAC (1 µg/mL) on the unbound fraction of FLU (20 µg/mL) was significant (14.04% increase) in HSA samples (40 g/L, Table 5); yet, it was still much lower than in samples containing pooled plasma (104.59%, Figure 1). A similar effect was seen with IMA (1 µg/mL) whereas the percent increase in unbound fraction of FLU (20 µg/mL) in pooled plasma, in HSA samples and in AGP samples were, respectively, 44.11% (Figure 1), 18.21% (Table 5), and 2.43% (Table 6). For 5-FU (1 µg/mL) the observed interactions in the presence of FLU, both in pooled plasma and HSA solutions, were less pronounced than with PAC and IMA whereas the increase in the unbound fraction of FLU (20 µg/mL) was by 5.97% (pooled plasma, Figure 1) and 9.32% (HSA samples, Table 5). This minor effect, compared to PAC and IMA goes along with its low protein binding (8–12%) [63].

Even though the observed increase in FLU unbound fraction in the presence of IMA was significant, higher increases could have been expected with IMA, which like PAC is highly bound to plasma proteins (95%) [54]. The lower displacing effect of PAC and IMA on unbound FLU seen in pooled plasma, could be explained as follows: (1) IMA is mainly bound to AGP [57], while PAC is mainly bound to HSA, (2) pooled plasma contains other endogenous/exogenous compounds that could also affect the binding of FLU or influence the effects exerted by PAC and IMA on FLU, this is supported by the similar effects obtained with HSA samples containing pure HSA, FLU and IMA/PAC, and (3) a difference in the affinity of IMA to plasma proteins. This latter one can be supported by the observed increase in unbound FLU in the presence of warfarin and diazepam frequently used as site I and site II displacing markers. As both drugs are around 99% bound [64,65], displacing effects similar or higher to those seen with PAC and IMA were expected. Interestingly, when tested at the same concentrations as IMA and PAC, the increase in the unbound fraction of FLU (20 µg/mL) were less than expected [14.37% (warfarin) and 20.04% (diazepam)] (Table 4). Consequently, it seems that both agents, show different affinity to HSA and weren’t able to displace FLU similar to PAC and IMA. The Sudlow’s Site I of HSA is able to simultaneously bind a wide variety of structurally different ligands, yet, with poor stereo-selectivity; even the binding of fatty acids influences the tertiary structure and the binding of site I ligands [1]. Site II, on the other hand, is smaller and stereo-selective. It has been shown that displacement interactions with fatty acids due to the high affinity of FA3-FA4 binding sites are more likely to occur on this site [1]. The effects of PAC’ binding to HSA, associated with major conformational changes, unfolding of the structure and significant increases of the hydrodynamic volume of the PAC-HSA complex [66], support the assumption of possible allosteric interactions when co-administered with other drugs. Moreover, indications for 5-FU to cause conformational changes in HSA have already been documented [67]. Therefore, allosteric changes induced by PAC and 5-FU on HSA could be one cause for the detected DDI.

Interestingly, while previous data reported on the effect of various drugs on the metabolism of PAC [68,69] and IMA [43], unbound fraction of IMA [70], as well as the potency, toxicity, and deadly effects of 5-FU [55,66,71,72], this is the first report showing a significant effect of IMA, PAC, and 5-FU on the displacement of drugs such as FLU from plasma proteins under physiological conditions.

The interactions were also evaluated in conditions encountered in critically ill cancer patients. As these patients, suffer in many instances from additional pathological conditions affecting their kidney and liver, major routes for drugs’ metabolism and elimination. Reduced renal and hepatic functions leads to lower HSA levels (hypoalbuminemia) in elderly patients, which associates with decreased drug binding to HSA and eventually results in the accumulation of the administered drugs [1]. Inflammatory diseases and conditions such as surgery, burn and malnutrition can temporarily exacerbate this already present hypoalbuminemia [1]. The effect of hypalbuminemia on protein binding characteristics of some antibiotics, including FLU in critically ill patients has already been demonstrated, whereas increased unbound drug fraction was measured while the total drug concentration was within the reference range [73]. Moreover, the peak concentration of ceftriaxone, another antibiotic, has been shown to fall under the minimum effective concentration solely due to lower HSA concentration in patients with normal renal function [1].

Interestingly, there was no significant difference in the percentage of binding of FLU when spiked at two different concentrations into physiological (40 g/L)/pathophysiological (25 g/L) solutions of HSA (Figure 2). Even though our data are in contrast to those reported in real patients [13], the plausible explanation is that the samples in this study contained only FLU and HSA whereas in real patients other endogenous and exogenous molecules are present and also compete for the same binding sites of FLU.

Intriguingly, when FLU was combined with either of PAC, IMA, or 5-FU at pathophysiological levels of HSA, differential effects were observed. For instance, while hypoalbuminemia did not have a significant effect on the displacing effect of IMA, only at a ratio of 1:5 (FLU/5-FU) a significant increase in unbound FLU (11.18% (25 g/L HSA) vs. −3.19% (40 g/L HSA)) was observed. Similarly, in comparison to physiological levels of HSA, a 2.5-fold increase in the displacing effect of PAC was observed (Table 5).

In addition to hypoalbuminemia, pathological conditions such as arthritis, myocardial infarction, and cancer raise AGP levels thus enhancing the binding of drugs [27]. As expected, at both tested concentrations and in comparision to the binding of FLU to physiological AGP levels (0.9 g/L), more FLU was bound to increased levels of AGP (2.5 g/L) (Figure 2).

## 4. Materials and Methods

### 4.1. Standards and Reagents

IMA, PAC, 5-FU, and methanol, were obtained from Sigma-Aldrich (Munich, Germany). HSA and AGP were obtained from Sigma-Aldrich (Steinheim, Germany). FLU sodium salt was purchased from Toronto Research Chemicals (Toronto, ON, Canada). Flucloxacillin-13C4-14N sodium salt (FLU-13C4-14N) was from Alsachim (Illkirch, France). Sterile water was purchased from B. Braun Melsungen AG (Melsungen, Germany). All reagents used were of the highest available analytical grades. Drug free human plasma was obtained from the Blood Donors’ Center in Regensburg (Haema, Regensburg, Germany).

### 4.2. Preparations of Stock Solutions, Standards, and Quality Control Samples

An individual first set of stock was prepared for FLU in water. The stock was used to prepare the standards (SD) and quality controls (QC) on each run day. The SD and QC were diluted in water to generate the appropriate concentrations. The SD and QC were diluted in pooled plasma on each lab day to generate the required concentrations. The percentage of water in the working solutions did not exceed 10% to maintain the integrity of the plasma. The concentration of FLU in the plasma standards covered the expected range in human plasma (0.1 to 40 µg/mL) [19,74]. The QC with the highest concentration (QCH) was prepared at a concentration of 20 µg/mL and further diluted to obtain the low QC (QCL) at a concentration of 0.2 µg/mL. All samples were stored at −80 °C. Pooled plasma was used to determine, in vitro, the protein binding of FLU to human plasma proteins. HSA stocks, prepared by dissolving fraction V albumin powder in water, were further diluted to yield stocks of 25 g/L and 40 g/L. AGP stocks were prepared in water and used to generate 0.9 g/L and 2.5 g/L stocks. FLU was spiked, at two different concentrations (0.2 and 20 µg/mL), to plasma and the different stocks of HSA and AGP.

### 4.3. FLU Determination by LC-MS/MS

The analysis was conducted using an already established liquid chromatography tandem mass spectrometry (LC-MS/MS) method [74] after slight modifications. A 1200 series liquid chromatography system (Agilent, Waldbronn, Germany) consisting of G1312B binary pump, CTC-PAL Autosampler (CTC Analytics AG, Zwingen, Switzerland) and G1316B column oven module, connected to an API 4000 triple-quadrupole mass spectrometer equipped with electrospray (ESI) source (Applied Biosystems/Sciex, Darmstadt, Germany), was used for the analysis of the prepared samples. For the chromatographic separation, a reversed phase Kinetex F5 column with TMS endcapping (50 mm × 2.1 mm, 2.6 µm) (Phenomenex, Aschaffenburg, Germany) and an UltraLine UHPLC In-Line Filter (In-Line Assembly with Filter) (Restek, Bellefonte, PA, USA) was used. The temperature of the column was set at 50 °C, while the auto sampler tray temperate was set at 8 °C. The mobile phases, water (MPA) and methanol (MPB) charged with 2 mmol/L ammonium acetate and 0.1% formic acid, were delivered using a binary pump. After injecting fifteen µl into the column, a gradient elution at a flow rate of 0.5 mL/min was used to elute the components. From start to 0.15 min the mobile phase consisted of 96% MPA. MPB was linearly increased to 55% (from 0.15 to 1.7 min) and further increased to 100% MPB (from 1.7 to 2.3 min). MPB was retained at 100% from 2.3 to 3.3 min. The analytical column was re-equilibrated to initial conditions at 3.3 min. The total run time was 4 min. Isotopically labelled internal standard was used for FLU. Multiple reactions monitoring (MRM) in positive mode was used for the detection of the eluting compounds. The MS/MS instrument was operated with the following parameters: capillary voltage (5.5 kV)**,** desolvation gas (nitrogen) heated at 400 °C.The dwell time for all the transitions was 10 ms. Details of the MRM-transitions, collision energy, de-clustering potential, and exit potential of FLU and its internal standard are detailed previously [74]. Data acquisition and peak integration were performed using Analyst software (1.6.2) (Applied Biosystems/Sciex, Darmstadt, Germany). Total FLU was analyzed using isotopically labeled internal standard (IS), while analysis of unbound FLU was performed without IS. When used, IS was prepared fresh on each day using ice-cold methanol. Protein binding values were determined by subtracting the unbound values from the total FLU values in total plasma/HSA/AGP.

### 4.4. Sample Preparation for Total and Unbound FLU

Spiked samples were kept at 37 °C for 30 min and mixed with agitation (350 rpm) to allow the equilibrium between unbound and bound drug. For the analysis of unbound FLU, Amicon Ultra-0.5 centrifugal filter devices with a molecular weight cutoff of 30 kDa were used for the ultrafiltration of the samples. Briefly, 250 µL of spiked samples was inserted into the Amicon filter and centrifuged at 14,000× *g* for 30 min at 20 °C. To fit with the concentration ranges of the established method, the collected filtrates were further diluted in the auto-sampler vials before injection into the LC-MS/MS system. Extraction and analysis of total FLU from spiked samples were processed as follows: 40 µL were extracted (1:5 *v*/*v*) with internal standard working solution (ISWS), vortexed for 3 min and centrifuged for 5 min at 13,000× *g* at 4 °C. Samples were further diluted in water (1:5 *v*/*v*) in auto-sampler vials. Fifteen µL from all samples (total and unbound) was injected into the chromatographic system.

### 4.5. Statistics

All data were obtained from independent days from at least four replicates per each sample condition. Statistical significance for the difference in FLU unbound values was evaluated using student t-test. *p* < 0.05 was considered significant.

## 5. Conclusions

Highly bound drugs with narrow therapeutic index are prone to clinically important displacement interactions especially in presence of liver and/or kidney impairments, often encountered in ICU, and which result in an increase or a decrease in the drugs’ pharmacological effects. For instance, as a time-dependent β-lactam antibiotic a faster elimination due to an increase in the unbound fraction of FLU can be detrimental for the patients. Consequently, preservation of the unbound drug levels above the bacterial minimum inhibitory concentration during the dosing period is fundamental to achieve a positive clinical outcome. This study highlights two important findings. To the best of our knowledge this is the first report showing the significant binding of FLU to AGP and the interaction of FLU with IMA, PAC, or 5-FU, which can be clinically relevant following their co-administration. Furthermore, the fact that the selected anticancer drugs are used against different cancer types allows the generalization/application of the observed effects for a wide range of cancer patients. Further investigations and in vivo studies are needed to decipher the clinical relevance of the observed interactions. Meanwhile, with the ultimate aim of improving the clinical outcome of FLU therapy, therapeutic drug monitoring of the unbound fraction of FLU could be the key to assure its success when co-administered with the tested and other similar drugs. An exact observation of the free fraction is important, as changes in the percentage of plasma binding can influence the active concentration without influencing the total concentration; in this case dose adjustment can be made if any changes in its free fraction are asserted.

## Figures and Tables

**Table 1:** Interactions between FLU and PAC in pooled plasma. Data obtained from independent experiments each done with at least four replicates of each concentration. Abbreviations: AB conc = antibiotic concentration; AC conc = anticancer agent concentration; %CV = coefficient of variation. *** *p* < 0.001 with respect to FLU alone.

Sample	AB Conc (µg/mL)	AC Conc (µg/mL)	Measured Total Conc in Plasma (µg/mL)	%CV	Measured Unbound Conc in Plasma (µg/mL)	%CV	%Unbound
**FLU alone** **FLU alone**	0.2 20	- -	0.24 21.76	2.03 4.50	0.0016 0.1275	4.83 10.15	0.66 0.59
**FLU + PAC** **FLU + PAC**	0.2 0.2	0.2 2.0			0.0023 0.0030	3.64 2.87	0.96 *** 1.21 ***
**FLU + PAC** **FLU + PAC** **FLU + PAC** **FLU + PAC**	20 20 20 20	0.2 0.6 1.0 2.0			0.2211 0.2598 0.2609 0.2641	7.17 4.00 3.35 4.56	1.02 *** 1.19 *** 1.20 *** 1.21 ***

**Table 2:** Interactions between FLU and IMA in pooled plasma. Data obtained from independent experiments each done with at least four replicates of each concentration. AB conc = antibiotic concentration. AC conc = anticancer agent concentration. %CV = coefficient of variation. * *p* < 0.05, ** *p* < 0.01, *** *p* < 0.001 with respect to FLU alone at 20 µg/mL and 0.2 µg/mL.

Sample	AB Conc (µg/mL)	AC Conc (µg/mL)	Measured Total Conc in Plasma (µg/mL)	%CV	Measured Unbound Conc in Plasma (µg/mL)	%CV	%Unbound
**FLU alone** **FLU alone**	0.2 20	- -	0.23 21.18	2.7 11.3	0.0016 0.21	12.83 6.64	0.7 0.98
**FLU + IMA** **FLU + IMA**	0.2 0.2	0.2 2.0			0.0021 0.0023	7.69 6.61	0.89 * 1.0 **
**FLU + IMA** **FLU + IMA** **FLU + IMA** **FLU + IMA**	20 20 20 20	0.2 0.6 1.0 2.0			0.26 0.27 0.30 0.31	4.89 6.45 11.57 4.55	1.22 *** 1.27 *** 1.41 ** 1.47 ***

**Table 3:** Interactions between FLU and 5-FU in pooled plasma. Data obtained from independent experiments each done with at least four replicates of each concentration. AB conc. = antibiotic concentration. AC conc. = anticancer agent concentration. %CV = coefficient of variation. * *p* < 0.05, ** *p* < 0.01 with respect to FLU alone at 20 µg/mL and 0.2 µg/mL.

Sample	AB Conc. (µg/mL)	AC Conc. (µg/mL)	Measured Total Conc. in Plasma (µg/mL)	%CV	Measured Unbound Conc. in Plasma (µg/mL)	%CV	%Unbound
**FLU alone** **FLU alone**	0.2 20	- -	0.28 23.09	7.48 2.84	0.0023 0.1766	5.23 9.57	0.78 0.76
**FLU + 5-FU** **FLU + 5-FU**	0.2 0.2	0.2 2.0			0.0025 0.0026	4.54 1.68	0.88 ** 0.91 **
**FLU + 5-FU** **FLU + 5-FU** **FLU + 5-FU** **FLU + 5-FU**	20 20 20 20	0.2 0.6 1.0 2.0			0.1777 0.1832 0.1871 0.2026	11.6 10.1 3.00 1.60	0.77 0.79 0.81 0.88 *

**Table 4:** Interaction between FLU and warfarin (WAR)/or diazepam (DIA) in pooled plasma. Data obtained from independent experiments each done with at least four replicates of each concentration. % difference (% Diff) calculated with respective to the % unbound (% Unb) values of FLU alone at 20 µg/mL and 0.2 µg/mL. AB conc = antibiotic concentration. %CV = coefficient of variation. * *p* < 0.05, with respect to FLU alone.

Sample	AB Conc (µg/mL)	Drug Conc (µg/mL)	Measured Total Conc in Plasma (µg/mL)	%CV	Measured Unbound conc in Plasma (µg/mL)	%CV	% Unb	% Diff
**FLU alone**	20		20.722	2.03	0.152	10.42	0.73	
**FLU + WAR**	20	1.0			0.173	5.91	0.84	14.37 *
**FLU + DIA**	20	1.0			0.182	12.01	0.88	20.04 *

**Table 5:** Interactions between FLU and PAC, IMA and 5-FU at physiological (40 g/L) and pathophysiological (25 g/L) levels of albumin. Data obtained from independent experiments each done with at least 4 replicates of each concentration. % difference (%Diff) calculated with respective to the % unbound (% Unb) values of FLU alone at 20 µg/mL and 0.2 µg/mL. ALB = albumin. AB conc = antibiotic concentration. AC conc = anticancer agent concentration. %CV = coefficient of variation. * *p* < 0.05, ** *p* < 0.01, *** *p* < 0.001 with respect to FLU alone.

Sample	AB Conc (µg/mL)	AC Conc (µg/mL)	Total Conc (µg/mL)	%CV	Unb Conc (µg/mL)	%CV	% Unb	% Diff
FLU alone FLU alone	0.2 20		0.25 19.03	10.44 3.54	0.002 0.173	3.37 7.68	0.84 0.91	
FLU in HSA (40 g/L) FLU in HSA (40 g/L)	0.2 20		0.197 16.04	4.78 5.96	0.011 0.966	8.97 5.50	5.61 6.02	
FLU in HSA (25 g/L) FLU in HSA (25 g/L)	0.2 20		0.232 18.214	6.37 3.15	0.012 1.182	14.75 3.53	5.09 6.51	
FLU in HSA (40 g/L) +PAC FLU in HSA (40 g/L) +PAC	0.2 20	1.0 1.0	0.20 16.04	3.78 6.73	0.013 1.102	11.45 3.93	6.59 6.87	16.84 * 14.04 ***
FLU in HSA (40 g/L) +IMA FLU in HSA (40 g/L) +IMA	0.2 20	1.0 1.0	0.20 16.04	3.78 6.73	0.0132 1.1426	2.96 4.30	6.81 7.12	20.91 ** 18.21 ***
FLU in HSA (40 g/L) +5-FU FLU in HSA (40 g/L) +5-FU	0.2 20	1.0 1.0	0.20 16.04	3.78 6.73	0.011 1.057	3.41 5.20	5.46 6.59	3.19 9.32 *
FLU in HSA (25 g/L) +PAC FLU in HSA (25 g/L) +PAC	0.2 20	1.0 1.0	0.22 18.47	4.17 1.07	0.016 1.341	4.94 1.02	7.18 7.26	41.62 *** 11.59 ***
FLU in HSA (25 g/L) +IMA FLU in HSA (25 g/L) +IMA	0.2 20	1.0 1.0	0.244 17.880	2.42 4.47	0.0153 1.3930	2.56 4.28	6.27 7.79	23.64 *** 19.71 ***
FLU in HSA (25 g/L) +5-FU FLU in HSA (25 g/L) +5-FU	0.2 20	1.0 1.0	0.22 18.47	4.17 1.07	0.012 1.229	4.23 2.89	5.64 6.66	11.18 * 2.26

**Table 6:** Interactions between FLU and IMA at physiological (0.9 g/L) and pathophysiological (2.5 g/L) levels of α-1-acid glycoprotein. Data obtained from independent experiments each done with at least 4 replicates of each concentration. % difference (%Diff) calculated with respective to the % unbound (% Unb) values of FLU alone at 20 µg/mL and 0.2 µg/mL. AGP = α-1-acid glycoprotein. AB conc = antibiotic concentration. AC conc = anticancer agent concentration. %CV = coefficient of variation. * *p* < 0.05, *** *p* < 0.001 with respect to FLU alone.

Sample	AB Conc (µg/mL)	AC Conc (µg/mL)	Total conc (µg/mL)	%CV	Unb Conc (µg/mL)	%CV	% Unb	% Diff
**FLU in AGP (0.9 g/L)** **FLU in AGP (0.9 g/L)**	0.2 20		0.261 21.060	1.17 6.01	0.1019 4.6563	3.47 1.42	39.08 22.11	
**FLU in AGP (2.5 g/L)** **FLU in AGP (2.5 g/L)**	0.2 20		0.266 20.745	4.60 4.50	0.0852 4.2773	3.90 2.07	32.04 20.62	
**FLU in AGP (0.9 g/L) + IMA** **FLU in AGP (0.9 g/L) + IMA**	0.2 20	1.0 1.0	0.261 21.060	1.17 6.01	0.1243 4.7695	3.23 1.37	47.69 22.65	22.05 *** 2.43 *
**FLU in AGP (2.5 g/L) + IMA** **FLU in AGP (2.5 g/L) + IMA**	0.2 20	1.0 1.0	0.266 20.745	4.60 4.50	0.1078 4.4688	5.36 2.50	40.55 21.54	26.56 * 4.47 *
